# Light and ripening-regulated BBX protein-encoding genes in *Solanum lycopersicum*

**DOI:** 10.1038/s41598-020-76131-0

**Published:** 2020-11-06

**Authors:** Bruno Silvestre Lira, Maria José Oliveira, Lumi Shiose, Raquel Tsu Ay Wu, Daniele Rosado, Alessandra Cavalcanti Duarte Lupi, Luciano Freschi, Magdalena Rossi

**Affiliations:** 1 Departamento de Botânica, Instituto de Biociências, Universidade de São Paulo, Rua do Matão, 277, São Paulo, 05508-090 Brasil; 2 Present Address: Cold Spring Harbor Laboratory, 1 Bungtown Road, Cold Spring Harbor, NY 11724 USA

**Keywords:** Plant physiology, Light responses

## Abstract

Light controls several aspects of plant development through a complex signalling cascade. Several B-box domain containing proteins (BBX) were identified as regulators of *Arabidopsis thaliana* seedling photomorphogenesis. However, the knowledge about the role of this protein family in other physiological processes and species remains scarce. To fill this gap, here BBX protein encoding genes in tomato genome were characterised. The robust phylogeny obtained revealed how the domain diversity in this protein family evolved in Viridiplantae and allowed the precise identification of 31 tomato SlBBX proteins. The mRNA profiling in different organs revealed that *SlBBX* genes are regulated by light and their transcripts accumulation is directly affected by the chloroplast maturation status in both vegetative and fruit tissues. As tomato fruits develops, three *SlBBXs* were found to be upregulated in the early stages, controlled by the proper chloroplast differentiation and by the PHYTOCHROME (PHY)-dependent light perception. Upon ripening, other three *SlBBXs* were transcriptionally induced by RIPENING INHIBITOR master transcriptional factor, as well as by PHY-mediated signalling and proper plastid biogenesis. Altogether, the results obtained revealed a conserved role of *SlBBX* gene family in the light signalling cascade and identified putative members affecting tomato fruit development and ripening.

## Introduction

Zinc finger transcription factors (TFs) comprise one of the most important families of transcriptional regulators in plants and play a central role in plant growth and development regulation, as well as in biotic and abiotic stress responses^1,2^. Among these TFs, B-box domain containing proteins (BBX) belong to a subclass characterised by the presence of one or two zinc finger B-box domains, which are predicted to be involved in protein–protein interactions^3^. BBX proteins were classified into five structure groups, according to the number of B-box and CCT (CONSTANS, CONSTANS-like and TIMING OF CAB1) domains and VP (valine-proline) motifs. Members of group I are characterised by the presence of two B-box domains in tandem, one CCT domain and one VP motif. Group II is similar to group I, also presenting two B-box domains and one CCT domain, but no VP motif. Group III contains a single B-box domain and a CCT. Group IV is characterised by the presence of two B-box domains but without CCT domain. Finally, group V is composed by proteins with just one B-box domain^3,4^. Although the VP is mentioned as a group I exclusive motif, it has already been identified in several proteins belonging to group III, IV and V; thus, the presence of the VP motif differs members from structure group I from II, but evidences show that it is not exclusive to the first^5^.

Out of the 32 BBX proteins identified in *Arabidopsis thaliana*, 21 have already been functionally characterised, being described as regulators of various processes such as seedling photomorphogenesis^6,7^, photoperiodic flowering regulation^8^, shade avoidance^9^, and responses to biotic and abiotic stresses^10^. Interestingly, 14 BBX proteins were also found to be components of the light signalling transduction pathway^4,6,11,12^, with 12 of them belonging to groups IV (8 proteins) and V (4 proteins). Four of the light-signalling group IV proteins act as positive regulators—AtBBX20^13^, AtBBX21^14^, AtBBX22^15^ and AtBBX23^16^—and the other four play a negative role—AtBBX18^17^, AtBBX19^18^, AtBBX24^19^ and AtBBX25^20,21^. In the case of group V, only repressors of light signal transduction were reported, AtBBX28^6^, AtBBX30^7^, AtBBX31^7^ and AtBBX32^22^.

BBX proteins act by the direct or indirect interaction with central components of the light signal transduction network, including the transcription factors ELONGATED HYPOCOTYL 5 (HY5), HOMOLOG OF HY5 (HYH) and PHYTOCHROME INTERACTING FACTORs (PIFs), and the protein-ubiquitin ligase CONSTITUTIVE PHOTOMORPHOGENIC1 (COP1)^4,23^. For instance, AtBBX21 and AtBBX22 promote HY5 transcript accumulation and can be tagged for proteasomal degradation via COP1-mediated ubiquitination^14,24,25^. In contrast, AtBBX24 and AtBBX25 downregulate light signalling by the physical interaction with HYH and HY5^20,26^. Interestingly, AtBBX28 was characterised as a light-induced light repressor, as it physically represses HY5 transcriptional regulatory activity and is marked for degradation in darkness by COP1^6^. Yet, it was demonstrated that PIF3 and PIF1 transcription factors signalling cascade regulates *AtBBX23* transcription, whose product physically interacts with HY5 inducing photomorphogenesis in *A. thaliana* seedlings^12^.

The above-described links between BBXs and light signalling have been almost exclusively explored in seedling photomorphogenesis, and their role in other light-controlled physiological processes, such as plastid development and maintenance, plant architecture and fruit development, which are important determinants of crop yield and nutritional quality^27^, remains elusive. In this context, although the effect of light perception and signalling in tomato (*Solanum lycopersicum* L.) fruit productivity and nutraceutical composition has been increasingly demonstrated^28–35^, the association of the BBX protein family with light in this species is still elusive. In tomato, 29 BBX domain encoding genes were identified and reported to be modulated by abiotic stress and phytohormones^36^. Additionally, the Solyc01g110180 locus encodes the only deeply characterised tomato BBX, which is a positive regulator of fruit carotenogenesis^37^.

Here, a comprehensive genome survey allowed the identification of 31 BBX protein-encoding loci in tomato genome. A robust phylogenetic reconstruction corroborated the monophyletic nature of the five previously identified structure groups and allowed the proposition of a new interpretation of the evolutionary history of this protein family. Further, we focused on the transcriptional profile of the 15 genes belonging to groups IV and V, revealing their association with organ greening and light signalling. Additionally, six genes were either up- or downregulated from immature fruit stages towards ripening. Finally, it was addressed whether the mRNA accumulation of these six genes is regulated by PHYTOCHROME (PHY)-mediated light perception and/or plastid development and differentiation.

## Materials and methods

### Plant material, growth conditions and sampling

Different tomato (*Solanum lycopersicum* L.) cv. Micro-Tom genotypes were used for *SlBBXs* transcriptional analysis: control genotype harbouring the wild-type *GOLDEN-2 LIKE 2 (SlGLK2)* allele (WT)^38^; uniform ripening *Slglk2* mutant, which is deficient in SlGLK2, the master transcription factor controlling fruit chloroplast differentiation and maintenance^33^ and; fruit-specific transgenic lines silenced for *SlPHYA* (*SlphyA*) and *SlPHYB2* (*SlphyB2*)^30^. Although Micro-Tom cultivar is deficient in brassinosteroid biosynthesis due to the weak mutation *dwarf (d)*, it has been extensively demonstrated that represents a convenient and adequate model system to study fruit biology^39^. In this work we used Micro-Tom variety because we have all the germplasm collection in this background, including *Slglk2* mutant and the fruit-specific *SlPHY*-silenced transgenic lines.

For the experiments with seedlings, seeds were in vitro germinated in the darkness as described in^40^. After 2 days, seedlings were either kept in the darkness or transferred to the light (12 h photoperiod) for another 7 days, when hypocotyls and cotyledons were sampled.

Leaves and fruits were harvested from plants cultivated in 2L rectangular plastic pots containing a 1:1 mixture of substrate and vermiculite supplemented with NPK 10:10:10, dolomite limestone (MgCO_3_ + CaCO_3_) and magnesium thermophosphate (Yoorin), under controlled temperature (between 23 °C and 27 °C), daily automatically irrigation by capillarity, and under natural light conditions (13 h photoperiod and 250–350 μmolm^−2^ s^−1^ of incident photo-irradiance) in a biosafety level 1 greenhouse.

Source and sink leaves were harvested from 4 and 8th phytomer closest to the base of the plant, respectively, of plants with 40-day-old plants^34^. Fruit pericarp, without placenta and locule walls, was collected from fruits at different stages: (i) immature green 3 (IG3, approximately 8 days post-anthesis); (ii) immature green 5 (IG5, approximately 15 days post-anthesis); (iii) mature green (MG, when the placenta displays a gelatinous aspect, approximately 26 days post-anthesis); (iv) breaker (Br, beginning of ripening process when the fruit begins to present a yellowish coloration, approximately 32 days post-anthesis); (v) Br3 (three days after breaker stage, the fruits presents orange coloration); (vi) Br5 (5 days after breaker stage). Fruits were sectioned in three parts: (i) pedicellar, also known as the green shoulder, where developed chloroplast are predominately located, (ii) stylar region, which lacks developed chloroplasts), and (iii) the middle region that was discarded. For all the experiments, at least four pools of fruits (biological replicates) were harvested from at least five plants. Samples were frozen in liquid nitrogen and stored at − 80 °C freezer until processing. Mature green fruits were used for chromatin immunoprecipitation assay.

### Phylogenetic analysis

For phylogenetic analysis BBX proteins from plant species representing angiosperms and Chlorophyta, as well as from Homo sapiens (as outgroup) were used. The loci encoding BBX proteins were retrieved from: Phytozome 12.1 (https://phytozome.jgi.doe.gov) database for *Arabidopsis thaliana, Chlamydomonas reinhardtii, Solanum lycopersicum* and *Volvox carteri* and, from NCBI ref-seq database (https://www.ncbi.nlm.nih.gov/refseq/) for *Chlorella variabilis, Coccomyxa subellipsoidea C-169, Homo sapiens, Micromonas commode, Micromonas pusilla CCMP1545, Ostreococcus lucimarinus CCE9901*, *Ostreococcus tauri* and *Volvox carteri f. nagariensis* (Supplementary Table S1).

Sequences from *A. thaliana*^3^ and tomato^36^ were named as previously reported. Amino acid sequences were aligned with Expresso T-COFFEE^41^ and the phylogeny was reconstructed as described in^42^. Briefly, the protein alignment was subjected to maximum likelihood phylogenetic reconstruction (PHYML 3.0) by JTT model with the proportion of invariable sites and gamma shape parameter estimated from the data sample. The obtained tree was optimized by tree topology and branch length, improved by subtree pruning and regrafting, and the branch support was calculated by the approximate likelihood-ratio test Shimodaira-Hasegawa-like (aLTR SH-like).

### Reverse transcriptase quantitative PCR analysis (RT-qPCR)

RNA extraction, complementary DNA (cDNA) synthesis, primer design and RT-qPCR assays were performed as described by^43^. Primer sequences used are detailed in Supplementary Table S2. qPCR reactions were carried out in a QuantStudio 6 Flex Real-Time PCR system (Applied Biosystems) using 2X Power SYBR Green Master Mix reagent (Life Technologies) in a 10 µL final volume. Absolute fluorescence data were analysed using the LinRegPCR software package^44^ in order to obtain quantitation cycle (Cq) values and calculate PCR efficiency. Expression values were normalised against the geometric mean of two reference genes, *TIP41* and *EXPRESSED*, according to^43^. A permutation test lacking sample distribution assumptions^45^ was applied to detect statistical differences (*P* < 0.05) in expression ratios using the algorithms in the fgStatistics software package version 17/05/2012^46^.

### Chromatin immunoprecipitation assay (ChIP)

Full‐length cDNA encoding RIPENING INHIBITOR transcription factor (*SlRIN*, Solyc05g012020) without the stop codon was amplified with the primers listed in Supplementary Table S2. The fragment was cloned into pENTR/DTOPO using Gateway technology (Invitrogen). The entry plasmids were recombined into pK7FWG2^47^ using LR Clonase (Invitrogen) to produce 35S::SlRIN-GFP fusion protein. The construct obtained was introduced into *Agrobacterium tumefaciens* (GV3101) for further infiltration. ChIP assay followed by qPCR was performed as described in^34^. Briefly, MG fruits were agroinfiltrated with *35S::SlRIN-GFP* construct, kept for 3 days under 16 h/8 h photoperiod, and fixed with formaldehyde to promote the cross-linking between DNA and proteins. Following nuclei enrichment with a Percoll (GE Healthcare) gradient, the chromatin was fragmented by sonication (10 s on/20 s off, amplitude 70, during 10 min using QSonica700 device) and then incubated with Dynabeads Protein‐A (Invitrogen) with either anti-GFP or anti‐HA antibodies (Invitrogen). Next, the immunoprecipitated DNA was purified by phenol:chloroform:isoamyl alcohol extraction and used as template for qPCR analysis. Specific primer pairs flanking the predicted TF binding motif for each promoter region and the coding region of *SlACTIN4* gene^48^ as control non-binding region (Supplementary Table S2) were used.

### Data analyses

Differences in parameters were analysed using Infostat software version 17/06/2015^49^. When the data set showed homoscedasticity, Student’s *t*-test (*P* < 0.05) was performed to compare transgenic lines against the control genotype. In the absence of homoscedasticity, a non-parametric comparison was performed by applying the Mann–Whitney test (*P* < 0.05). All values represent the mean of at least three biological replicates.

Transcription factor binding motifs were identified on the 3000 bp upstream of the transcription initiation site using PlantPAN 2.0^50^.

## Results

### *Solanum lycopersicum* harbours similar diversity of BBX protein-encoding genes than *A. thaliana*

The BBX TF family has been extensively studied in *A. thaliana*, whose proteins were classified into five groups accordingly to the domain structure^3,5^. Similar classification was reported for other species such as tomato^36^, potato^51^, rice^52^ and grapevine^53^. However, not all provided a phylogeny with high branch support for the groupings and the lack of outgroup led the evolutionary history of the protein family ambiguous.

To provide robust phylogenetic information, BBX domain-containing protein sequences from tomato and *A. thaliana* were retrieved from Phytozome database (https://phytozome.jgi.doe.gov) (Supplementary Table S1). This survey led to the identification of two additional loci encoding BBX proteins in the tomato genome, that were named *SlBBX30* and *SlBBX31*, following the previously nomenclature published for this species^36^. *A. thaliana* sequences were named according to the nomenclature adopted by^3^ (Supplementary Table S1).

**Figure 1:**
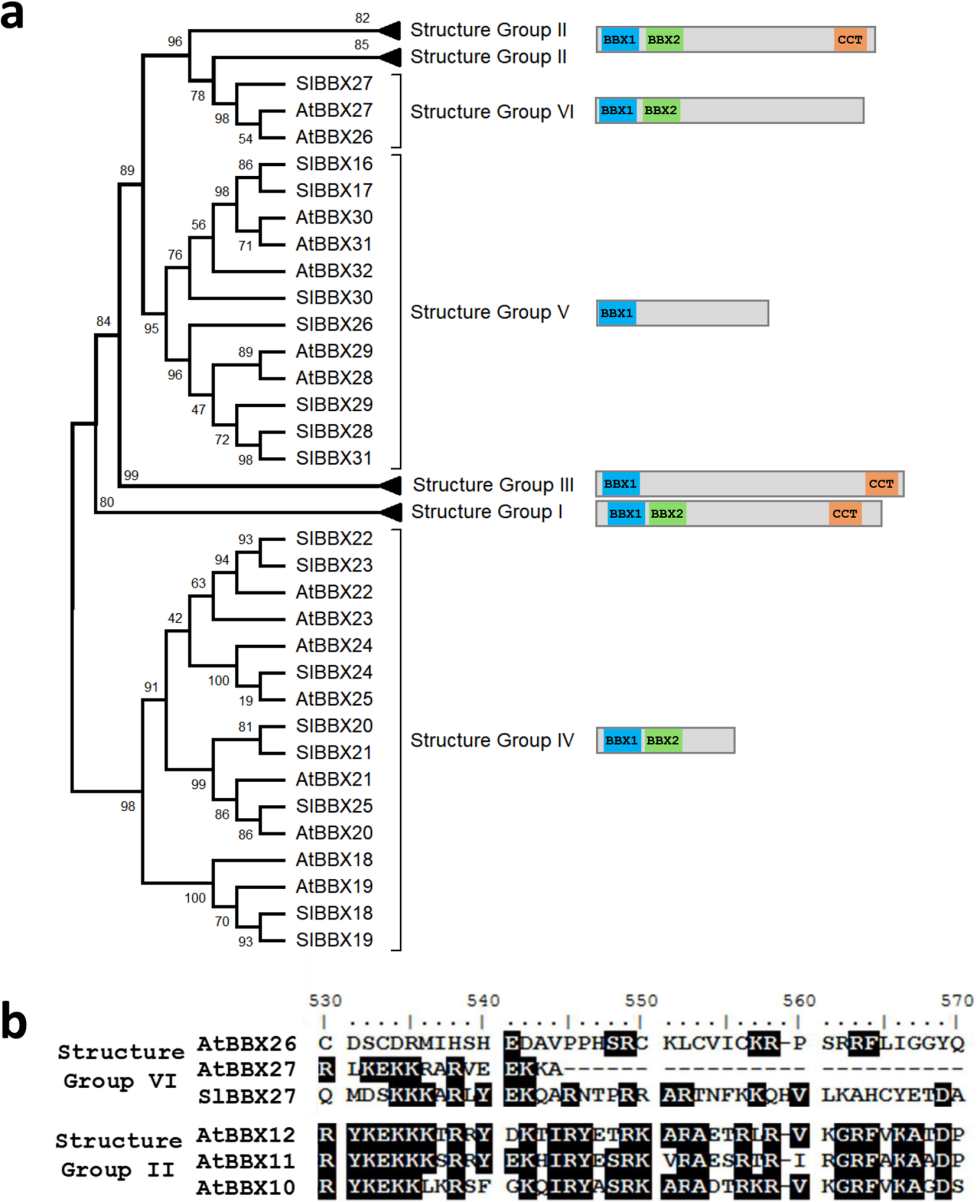
Phylogenetic presentation of *A. thaliana* and tomato BBX proteins. (**a**) Phylogenetic reconstruction obtained from the alignment of *A. thaliana* and tomato BBX proteins. The clusters were named accordingly to the structure groups described for A. thaliana and the domain architecture of each clade was determined using the consensus sequence. (**b**) A highlight of CCT motif alignment of structure group II representatives and the corresponding region of structure group VI sequences. Shading threshold = 60%.

The phylogenetic reconstruction (Fig. 1a) grouped the sequences according to their domain structure as previously reported in *A thaliana*^4^, confirming the monophyletic nature of the five structure groups. Regarding the tree topology, structure group IV appeared isolated from the other four groups, while groups II and V clustered together. Interestingly, AtBBX26 and AtBBX27 were previously classified in the structure group V^4^, while SlBBX27 was found clustered with group III proteins^36^; the three were described as a single B-box domain containing protein. Here, it was found that these three proteins contain indeed two BBX domains and grouped together as a subclade of structure group II without CCT domain, being referred as structure group VI (Fig. 1a). When the structure group VI sequences (i.e. AtBBX26, AtBBX27 and SlBBX27) and three representative sequences of structure group II (i.e. AtBBX10, AtBBX11 and AtBBX12) were aligned, the CCT motif could be clearly identified in the latter and some conserved residues could also be found in structure group VI sequences (Fig. 1b). Thus, this result indicates that the structure group VI diverged from structure group II sequences that lost the CCT motif.

The above described topology is in agreement with the one obtained for grapevine^53^, but is not with two other well supported phylogenies^5,54^. The approach applied here differed from the previously reported in two methodological aspects: human (*H. sapiens*) B-box domain-containing proteins were obtained from NCBI ref-seq database (https://www.ncbi.nlm.nih.gov/refseq/) (Supplementary Table S1) and used as an outgroup in the analysis; and the structure-based multiple sequence alignment whose accuracy surpass sequence-based only packages was applied^41^.

**Figure 2:**
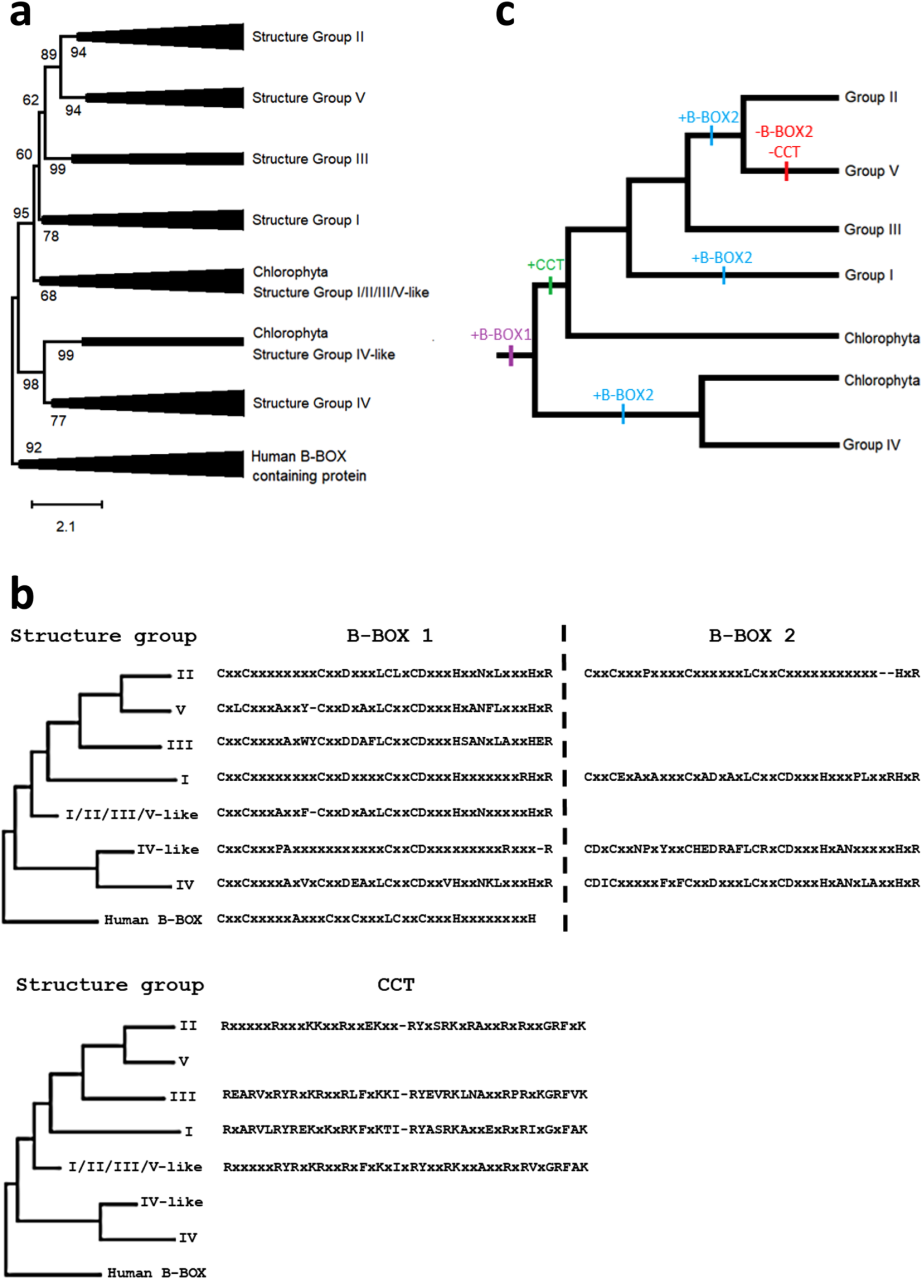
Evolution of BBX proteins. (**a**) Phylogenetic reconstruction obtained from the alignment of *A. thaliana,* tomato, chlorophyta and human B-box domain containing proteins. The clusters were named accordingly to the structure groups described for *A. thaliana*. The sequences information is available in Supplementary Table S1. (**b**) Consensus sequence for B-box and CCT domains (identity ≥ 60%). (**c**) Proposed hypothesis for domain evolution in the BBX protein family. While the B-box1 and CCT domains appear to have single origins along the evolution of these proteins, the B-box2 domain evolved independently three times.

Thus, to further confirm the obtained topology and bring information about the evolutionary history of this protein family, another phylogenetic analysis was performed including sequences from Chlorophyta species (Supplementary Table S1). The same above described topology for only tomato and *A. thaliana* was obtained. As the structure group VI was identified as a subclade of group II, group VI was collapsed with group II sequences to simplify the visualization (Fig. 2a). Moreover, two Chlorophyta clusters were observed, one grouping with the structure group IV and other with the clade composed of structure groups I/II/III/V. This indicated that the Viridiplantae ancestral, as means before the divergence of Chlorophyta and land plants, had two BBX-coding genes, one of which was subjected to three duplication events along land plants evolution.

The consensus sequence for the B-box and CCT domains was identified for each group (Fig. 2b). The CCT domain appear to have one single origin in the ancestral sequence of the structure groups I/II/III/V, before the divergence of Chlorophyta and land plants. It is not clear whether the ancestral proteins had one or two BBX domains. Based on the domain consensus, B-box1 seems to have a single origin, while B-box2 may have arisen several times independently, i.e. in the ancestral of the structure group IV clade, in structure group I group and in the ancestral of the structure groups II/V. Regarding the latter, the alignment of the sequences of both groups revealed that some B-box2 domain conserved residues could be still identified in structure group V members, however none could be identified in structure group III (Supplementary Fig. S1). Thus, this indicates that B-box2 appeared in the ancestral of structure group II and V after the divergence from group III. The occurrence of only B-box1 domain in structure group V is the consequence of the divergence of B-box2 and a deletion in the ancestral sequence that resulted in the loss of the CCT domain.

Concluding, these results bring evidences that the ancestral Viridiplanteae harboured two B-box containing proteins; the ancestral of group IV with two B-box domains and the ancestral of group I/II/III/V-like clade with a single B-box domain. This later, after the divergence of land plants and Chlorophyta, diverged into four structure groups in which B-box2 domain arose two times independently (Fig. 2c).

### The expression pattern of groups IV and V *SlBBX* genes is influenced by the stage of plastid development in both vegetative and fruit tissues

To gain insight into the link between BBX proteins and light signalling in tomato, we explored the transcription pattern of *SlBBX* genes that belong to the structure groups IV and V in organs bearing chloroplast at distinct light-regulated developmental stages, such as source and sink leaves, etiolated and de-etiolated seedlings and, fruits from immature to ripe stages^29,34,40^.

**Figure 3:**
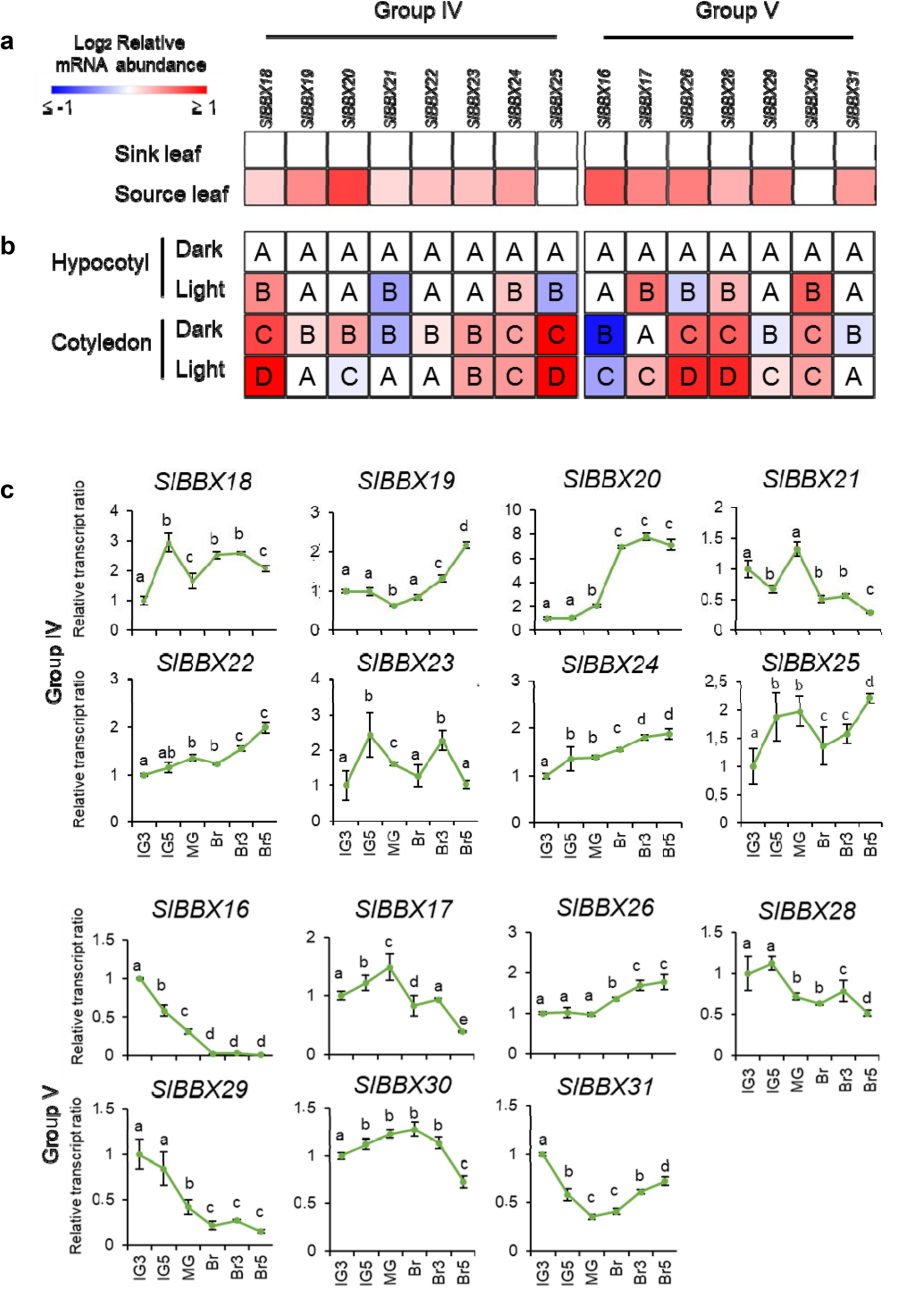
Transcript profile of structure group IV and V *SlBBX* genes. (**a**) Heatmap representation of the relative transcript ratio of *SlBBXs* in sink and source leaves from the 8th and the 4th phytomers of 40-day-old plants, respectively. Values are means of at least three biological replicates. Colored squares represent statistically significant differences in relation to the sink leaf sample (P < 0.05). Relative transcript values are detailed in Supplementary Table S3. (**b**) Heatmap representation of the relative transcript ratio of SlBBXs in etiolated and de-etiolated hypocotyls and cotyledons. Values are means of at least three biological replicates. Different letters represent statistically significant differences among the samples within each gene (P < 0.05). Relative transcript values are detailed in Supplementary Table S3. (**c**) Relative transcript ratio of SlBBXs in the pedicellar (top) portion throughout fruit development and ripening. Data were normalised against the IG3 sample. Values are means ± SE of at least three biological replicates. Different letters indicate statistically significant differences between fruit stages (P < 0.05). IG3: immature green 3; IG5: immature green 5; MG: mature green; Br: breaker; Br3: 3 days after Br; Br5: 5 days after Br.

As shown in Fig. 3a, *SlBBX* genes were significantly more expressed in source leaves than in sink counterparts, excepting *SlBBX25* and *SlBBX30* whose mRNA remained invariable. *SlBBX20* was the gene that showed the most expressive induction, approximately six times (Supplementary Table S3).

Transcript abundance of these *SlBBX* genes was also analysed under etiolation (skotomorphogenesis) and de-etiolation (photomorphogenesis) conditions in hypocotyls and cotyledons (Fig. 3b, Supplementary Table S3). Interestingly, most of the *SlBBX* genes showed higher levels of mRNA in cotyledons compared to hypocotyls, both in dark-grown (*SlBBX18*, *SlBBX19*, *SlBBX20*, *SlBBX22*, *SlBBX23, SlBBX24*, *SlBBX25*, *SlBBX26*, *SlBBX28* and *SlBBX30*) and light-grown (*SlBBX18*, *SlBBX21*, *SlBBX23, SlBBX24*, *SlBBX25*, *SlBBX26*, *SlBBX28* and *SlBBX29*) seedlings. Light exposure upregulated five (*SlBBX18*, *SlBBX24*, *SlBBX17*, *SlBBX28* and *SlBBX30*) and eight (*SlBBX18*, *SlBBX21*, *SlBBX16*, *SlBBX17*, *SlBBX26*, *SlBBX28*, *SlBBX29* and *SlBBX31*) genes in hypocotyls and cotyledons, respectively.

Finally, the transcript pattern of *SlBBXs* belonging to structure groups IV and V was profiled throughout fruit development and ripening. Since there is a chloroplast development gradient along the longitudinal axis in wild type (WT) tomato fruits^55^, they were sectioned in pedicellar (with more and more developed chloroplasts) and stylar (with less and poorly developed chloroplasts) portions. As the profiles from both sections were mostly similar (Supplementary Fig. S2), we focused the analysis on the pedicellar portion (Fig. 3c, Supplementary Table S4). Most *SlBBX* genes exhibited substantial variations in the mRNA accumulation within the analysed stages. Interestingly, six genes showed clear association with either early development or ripening of fruits: *SlBBX19* (Solyc01g110370), *SlBBX20* (Solyc12g089240) and *SlBBX26* (Solyc10g006750) were strongly upregulated upon ripening triggering, as means from MG to Br stage; while, the amount of *SlBBX16* (Solyc12g005750), *SlBBX28* (Solyc12g005660) and *SlBBX29* (Solyc02g079430) mRNA was higher at green stages of fruit development gradually declining afterwards. The most expressive fold changes were observed for *SlBBX20* and *SlBBX16*, which were eight times more and ten times less expressed from IG3 towards fully ripe Br5 fruits, respectively.

The comparison of the relative mRNA accumulation levels of groups IV and V *SlBBX* genes among all the four organs analysed displayed no evident organ or structural specificity; however, except for *SlBBX20* and *SlBBX22*, they showed the highest expression either in source leaves or cotyledons (Supplementary Fig. S3). To sum up, the results showed that the plastid type and developmental stage (i.e. proplastid, chloroplast or chromoplast) seem to affect the transcript accumulation pattern of these 15 *SlBBX* genes in leaves, hypocotyls, cotyledons and fruits.

### *SlBBX* genes associated with fruit early development or ripening are regulated by SlPHY and/or SlGLK2

The identification of *SlBBXs* whose transcript profile is associate with fruit development and the importance of plastidial metabolism for determining nutraceutical content of tomato fruit, led to the investigation whether SlGLK2, a transcription factor essential for fruit chloroplast differentiation and activity maintenance^33,55^, and PHY-mediated light perception^29^ participate in the transcriptional regulation of the six above highlighted *SlBBX* genes (i.e.* SlBBX16*, *SlBBX19*, *SlBBX20*, *SlBBX26*, *SlBBX28* and *SlBBX29*). The hypothesis that SlGLK2- and/or PHYs regulate these genes was reinforced by the finding, in their promoter regions, of at least one HY5 (key inductor of PHY-mediated photomorphogenesis^56,57^), PHYTOCHROME INTERACTING FACTORs (PIF; key repressor of PHY-mediated photomorphogenesis^58^), or GLK binding motifs^59^ (Supplementary Fig. S4). *Slglk2* mutant, which encodes a truncated and inactive version of the protein^55^, and two fruit-specific *SlPHY*-silenced transgenic genotypes were used for the mRNA profiling. Out of the five tomato PHYs^60^, fruit-specific functional characterization highlighted two as major contributors to fruit physiology: SlPHYA, a positive regulator of tomato plastid division machinery; SlPHYB2, a negative regulator of chlorophyll accumulation^30^ and; both, inductors of fruit carotenogenesis.

**Figure 4:**
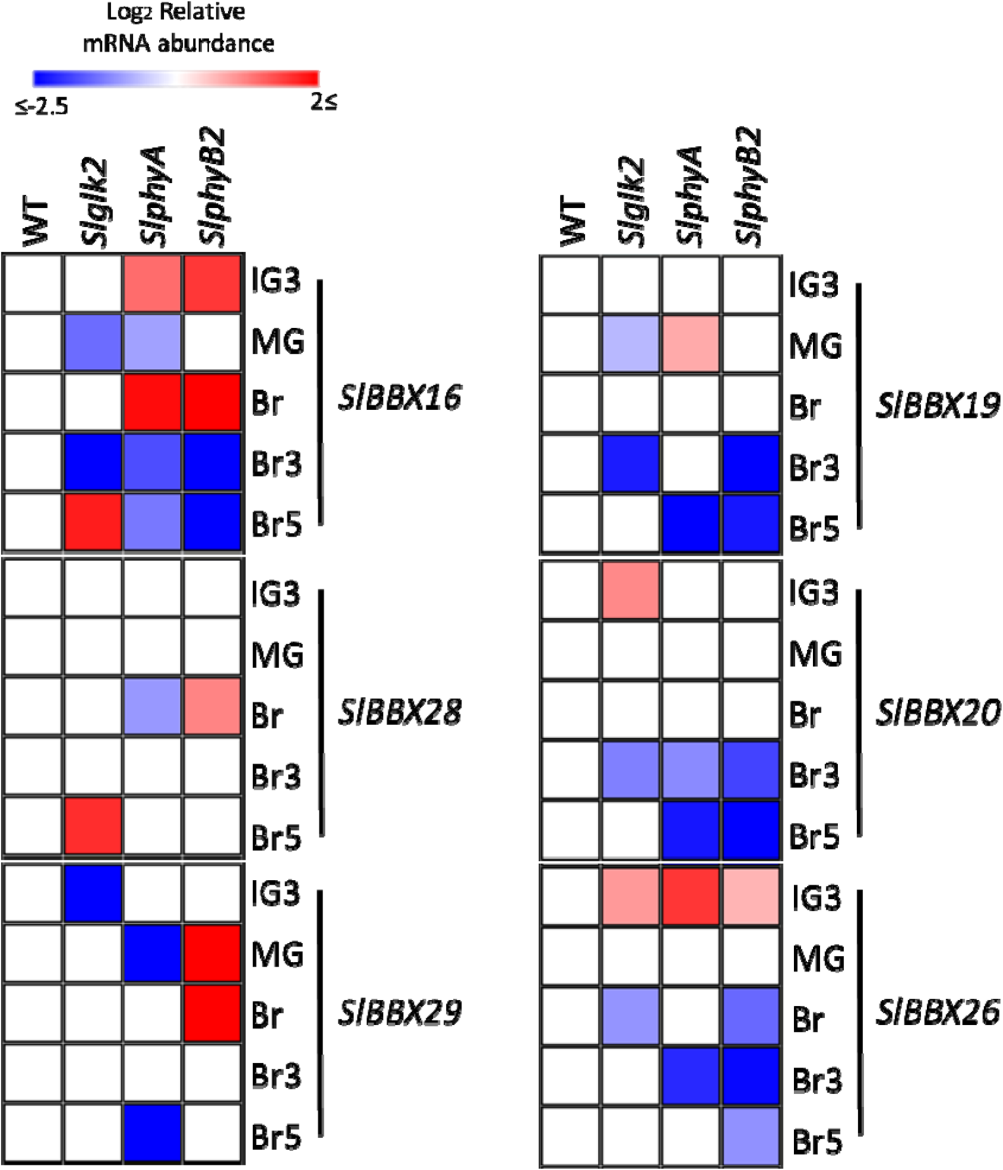
Transcriptional profile of *SlBBXs* in developing fruits of tomato lines impaired in light perception or chloroplast differentiation. The relative mRNA abundance of the six *SlBBXs* modulated by ripening was addressed in fruits of wild type plants (WT), SlGLK2-deficient mutant (*Slglk2*, Lupi et al. 2019), and fruit-specific *SlPHYA*- and *SlPHYB2*-silenced (*SlphyA *and *SlphyB2*) lines^30^. Values were normalised against the respective WT sample and are means of at least three biological replicates. The relative transcript values are detailed in Supplemental Table S5. Statistically significant differences relative to WT samples are colored (*P* < 0.05). IG: immature green 3; MG: mature green; Br: breaker; Br3: 3 days after Br; Br5: 5 days after Br.

Among the *SlBBX* genes downregulated during fruit development, *SlBBX28*, regardless punctual fluctuations, did not show clear pattern of SlPHY- and SlGLK2-dependent regulation (Fig. 4). In the case of *SlBBX29*, while the lack of SlGLK2 led to a reduced transcript amount at IG3; SlPHYs have opposite effects at MG stage. Yet, *SlBBX16* regulation appears to be more complex, at the peak of expression (i.e. IG3 stage) SlPHYA- and SlPHYB2-deficiency enhanced mRNA accumulation level. On the contrary, SlGLK2 seemed to have an inductive effect at green stages of fruit development (Fig. 4, Supplementary Table S5). The biological significance of the transcript level differences in the tested genotypes from Br to Br5 is questionable due to the extremely low amount of mRNA detected in ripening stages of WT genotype (i.e. the mRNA level of *SlBBX16* at Br stage is only 3% of the IG3 value, Supplementary Table S4).

The ripening induction observed in *SlBBX19*, *SlBBX20* and *SlBBX26* was attenuated in *SlPHYA*- and *SlPHYB2*-silenced fruits as well as in the SlGLK2-deficient genotype. This is clearly shown by the downregulation of their expression from Br towards Br5, suggesting that SlGLK2- and SlPHY-mediated signalling cascade stimulate the expression of these genes.

### RIPENING INHIBITOR (SlRIN) regulates ripening-dependent expression of *SlBBX*s

**Figure 5:**
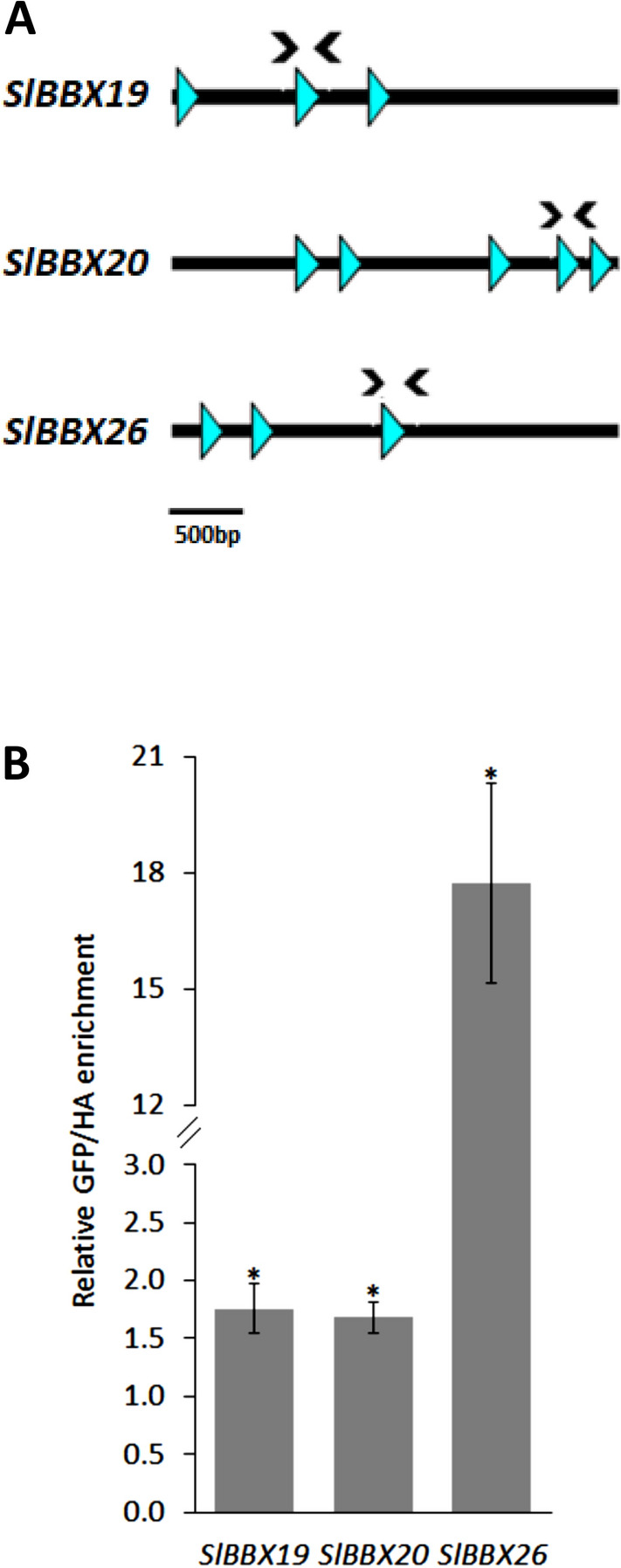
SlRIN binds to the ripening-induced *SlBBXs* promoter. (**A**) SlRIN binding motifs (C(CT)(AT)6(AG)G) blue triangles) in the promoter region (3000 bp upstream of the + 1 base) of the three ripening-induced *SlBBX* genes. Arrows indicate the positions of the primers used for ChIP‐qPCR assay. (**B**) ChIP‐qPCR experiment performed in tomato fruits transiently expressing *35S::SlRIN‐GFP* using anti‐GFP and anti‐HA (as negative control) antibodies. Asterisks denote statistically significant differences (*P* < 0.05) to the respective anti-HA sample.

SlRIN is a master TF controlling tomato fruit ripening^61^ whose binding motif C(CT)(AT)_6_(AG)G was identified after a genome wide ChIP-Seq experiment^62,63^. On the promoter region (3000 bp upstream the transcription initiation site) of the three ripening-induced *SlBBX* genes (i.e. *SlBBX19*, *SlBBX20* and *SlBBX26*), putative RIN binding motifs were identified (Fig. 5a). To address whether SlRIN directly interacts with the promoter of the aforementioned genes, a *35S::SlRIN‐GFP* construct was transiently expressed in WT mature green tomato fruits followed by a ChIP-qPCR assay with anti-GFP or negative control anti-HA antibodies. The anti-GFP immunoprecipitated chromatin showed to be enriched for all *SlBBX* promoters tested (Fig. 5b), demonstrating that SlRIN physically binds the regulatory region of *SlBBX19*, *SlBBX20* and *SlBBX26*, explaining the above-mentioned ripening-associated upregulation.

## Discussion

Over the past years, BBX protein family was surveyed in several species such as apple^64^, *A. thaliana*^4^, grapevine^53^, orchids^65^, pear^54^, rice^52^, potato^51^, *Arachis duranensis*^66^ and tomato^36^, being classified in five groups accordingly to the domain composition of the proteins. The comprehensive phylogenetic analysis performed in this work (Fig. 2a) provided evolutionary validation of this classification by revealing that the structure groups corresponded to well sustained monophyletic clusters. A foundational work^3^ performed a phylogenetic analysis of *A. thaliana* BBX protein family that was further revised by^5^, which proposed a model for BBXs evolutionary trajectory in green plants. Although the phylogeny topology obtained here does not reflect the evolutionary model proposed by^5^, two pieces of evidences showed by the phylogenetic analysis of B-box domains reported by these authors support the clustering observed here: (i) B-box2 domain from groups IV and I are more closely related than group II B-box2 and; (ii) B-box1 domain from groups II and V are the most closely related. Moreover, some methodological differences might have increased the accuracy of the topology obtained here: i) the incorporation of an outgroup; (ii) the multiple sequence alignment carried out with structure-based information^41^ and; (iii) the algorithm used for the multiple sequence alignment is consistency-based, whose accuracy is increased in comparison to matrix-based ones such as ClustalW^67^.

Our analysis showed that some *A. thaliana* and tomato proteins, previously reported as members of the structure group V^3,4^ and II^36^, respectively, are actually members of a new structure group, VI, which is diverging from group II after the loss of the CCT domain. As also observed for punctual examples belonging to groups II and V^4^, these results suggest that some BBX proteins lost a domain in a recent evolutionary event, but conserve other common characteristics of their structure group.

Concluding, based on phylogenetic and domain structure analyses, we propose that the ancestral Viridiplanteae harboured two B-box domain containing proteins that originated structure group IV-like and structure group I/II/III/V-like clades, respectively. Moreover, while B-box1 and CCT domains seem to single origins in the evolutionary history of this protein family, B-box2 arose three time, independently (Fig. 2c).

Functional studies regarding B-box domain encoding genes were performed almost exclusively in *A. thaliana* seedlings and, interestingly, especially members of structure group IV and V, were characterised as components of the light signalling cascade^13,14,16,18–21,24^. By employing different photoreceptors, plants can track light intensity, quality, periodicity and direction. Among photoreceptors, PHYs are codified by a small gene family, with members playing different roles gathering information for adjusting plant development and metabolism to the changing environment^68^. Once activated by light, PHYs phosphorylate several nuclear proteins controlling their function^69^. Among them, E3 ubiquitin ligase COP1 activity and stability is negatively modulated by PHYs^70^. Free of COP1 repression, the transcription factor HY5 is able to induce and repress the expression of photomorphogenesis- and skotomorphogenesis-related genes, respectively^57^. Several reports have pinpointed the major contribution of the above described light signal transduction pathway for determining tomato fruit yield and nutritional quality^30–35,71,72^. However, regarding *SlBBX* genes, only the locus Solyc01g110180, here named as *SlBBX25*, has been functionally characterised up to date, being described as a COP1-repressed positive regulator of chloroplast biogenesis, whose constitutive overexpression leads to dwarf plants bearing ripe fruits with increased carotenoid content^37^. Thus, it remains to be explored in a broader manner the role of BBX proteins in light-regulated physiological processes in tomato.

Here, in structure group IV and V, which encompasses most of the light-regulated BBX proteins described in *A. thaliana,* 15 tomato sequences were identified (Fig. 1). Then, they were transcriptionally profiled in source and sink leaves, seedling de-etiolation, and along fruit development and ripening (Fig. 3). The comparison of the mRNA accumulation level among the different profiled organs revealed that *SlBBX* transcripts accumulate most in source leaves or cotyledons (Supplementary Fig. S3), which is mostly in line with the profile previously reported in tomato^36^. The vast majority of *SlBBXs* displayed higher amounts of mRNA in source than in sink leaves hinting a correlation with chloroplast number and activity (Fig. 3a). The pattern of mRNA accumulation during seedlings skoto- and photomorphogenesis showed that out of the 15 analysed genes, 8 showed to be induced by light (*SlBBX16*, *SlBBX17*, *SlBBX18*, *SlBBX24*, *SlBBX28*, *SlBBX29*, *SlBBX30* and *SlBBX31*); while only four showed to be light-downregulated (*SlBBX19*, *SlBBX20*, *SlBBX22* and *SlBBX25*) in at least hypocotyl or cotyledon. Two genes showed inversed pattern in response to light in both organs (*SlBBX21* and *SlBBX26*) and one was invariable (*SlBBX23*). These results indicate that tomato BBX genes that belong to structure group IV and V are light responsive, like observed in *A. thaliana*^4^, and most are light-induced. The expression pattern of BBX encoding genes in *Solanum tuberosum* during de-etiolation was also addressed and the expression of most of the genes belonging to structural groups IV and V was modulated upon illumination of etiolated leaves^51^. This profile provides further evidences about a link between mRNA levels of BBX proteins from structure groups IV and V and plastid biogenesis and differentiation, revealing that they are affected, to some extent, by the light signalling cascade.

Regarding fruit development and ripening (Fig. 3c), six genes stood out as their transcripts were gradually reduced from green stages towards ripening (*SlBBX16, SlBBX28* and *SlBBX29*) or sharply induced upon this process triggering (*SlBBX19, SlBBX20* and *SlBBX26*), indicating that their expression is also modulated by the plastid developmental stage, i.e. chloroplast to chromoplast transition. Interestingly, with the exception of *SlBBX19* and *SlBBX26*, the mRNA accumulation profile observed here was in agreement with that reported by^36^.

Led by the particular pattern found in fruits for *SlBBX16*, *SlBBX19*, *SlBBX20*, *SlBBX26*,* SlBBX28* and *SlBBX29*, together with the occurrence in their promoter regions of binding motifs for TFs involved in the light signalling cascade (i.e. PIF, HY5 and GLK, Supplementary Fig. S4), their transcripts were profiled in genotypes with altered fruit light perception or without proper fruit chloroplast differentiation (Fig. 4). The three *SlBBX* genes downregulated from immature towards ripe stages showed induction by chloroplast maturation and light (Fig. 3a,b) and, except for *SlBBX28* that did not show alterations of its transcript abundance, *SlBBX16* and *SlBBX29* were induced in a SlGLK2- and SlPHY-dependent manner at green stages. SlGLK2, directly and/or indirectly, *i.e.* inducing chloroplasts biogenesis and maintenance^33,55^, promoted the mRNA accumulation of *SlBBX16* and *SlBBX29* at green stages of fruit development (Fig. 4). Interestingly, it was shown that SlPHYB2 represses *SlGLK2* mRNA accumulation^30^ thus, explaining the inducible effect of SlPHYB2 deficiency on the expression of these genes at green stages (Fig. 4). Finally, *SlPHYA*-silenced fruits displayed reduced number of chloroplasts with limited differentiation of its intermembranous structure^30^, which may be associated with the *SlBBX16* and *SlBBX29* downregulation detected in this genotype at MG stage.

The disruption of PHY-mediated light signalling or chloroplast differentiation by the lack of active SlGLK2 attenuated the ripening-associated transcript accumulation of *SlBBX19, SlBBX20* and *SlBBX26.* The minor effects observed in early stages indicate that these genes are rather induced along ripening than repressed during green stages of tomato fruit development. Since the mRNA amount of *SlGLK2* is almost undetectable from breaker towards fully ripe stage^33,55^, the observed reduction in mRNA level in *Slglk2* mutant for these three genes at ripening stages might be an indirect effect of the fewer and not fully differentiated chloroplasts in this genotype^33,55^, which are further converted into chromoplasts as ripening proceeds^73^. In a similar way, *SlPHYA*-silenced fruits also displayed poorly developed chloroplasts in the green stages^30^ that, as aforementioned, might lead to the observed reduction in the transcription of the three *SlBBX* genes. Interestingly, the observed downregulation of *SlBBX19* in the lack of PHYA or PHYB2 was also reported for its *A. thaliana* ortholog, *AtBBX19*, in *AtphyA* and *AtphyB* mutant seedlings^12^. As chlorophyll degrades, the chlorophyll self-shading effect is reduced allowing the pass of sunlight through the flesh of green fruit. Light shifts the photoequilibrium of PHYs to the active form promoting the inactivation of their downstream negative effectors SlPIFs and leading to the upregulation of light-dependent ripening associated genes^31,72^. As PIF-binding motifs were identified in *SlBBX19*, *SlBBX20* and *SlBBX26* promoters (Supplementary Fig. S4), these TFs that are altered in *SlphyA* and *SlphyB2*^30^ might downregulate the accumulation of these *BBX* transcripts in the PHY deficient lines.

Moreover, the ripening-associated mRNA accumulation of *SlBBX19, SlBBX20* and *SlBBX26* raised the hypothesis of the involvement of the master regulator of tomato fruit ripening SlRIN^61^ in the regulation of these genes. Indeed, in the promoter region of all three genes, RIN-binding motifs were found (Fig. 5a) and, by ChIP-qPCR, the direct binding of SlRIN was confirmed (Fig. 5b). This is in line with the previously reported ChIP-Seq results that showed the direct interaction between SlRIN and *SlBBX20* promoter^63^, and also with the reduced mRNA amount of this gene in *SlRIN*-silenced fruits^74^. Altogether, these results indicate that *SlBBX19, SlBBX20* and *SlBBX26* are light- and SlRIN-regulated, playing a role in tomato fruit ripening.

**Figure 6:**
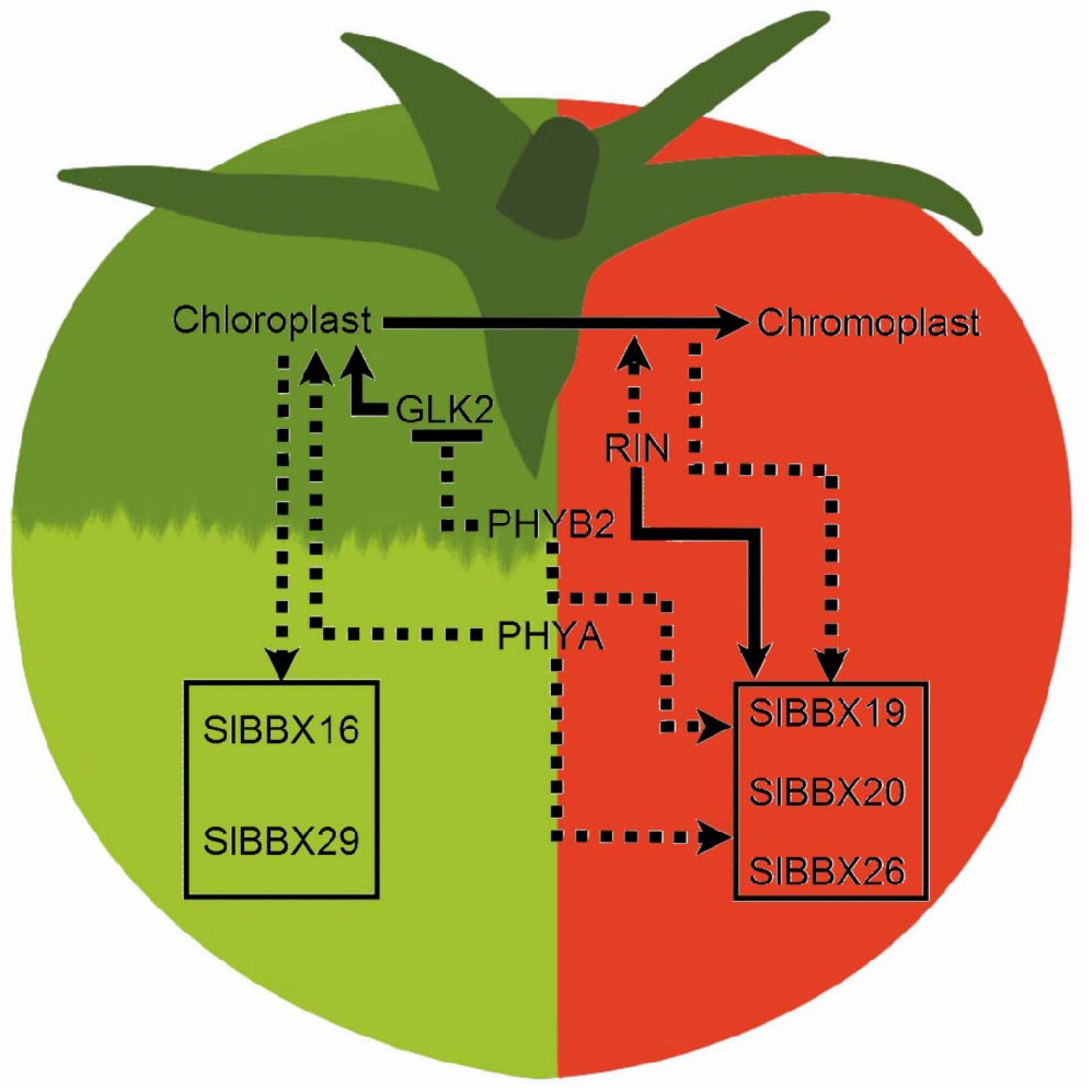
Proposed regulatory network for the control of fruit development- and ripening-associated *SlBBX* genes. During early tomato fruit development, SlGLK2 induces the expression of several genes leading to chloroplast differentiation. SlPHYs have an inverse effect over plastidial development at green stages. While SlPHYB2 inhibits SlGLK2 transcript accumulation, SlPHYA positively controls chloroplast division regulators^30^. Chloroplast biogenesis and maturation positively influence *SlBBX16* and *SlBBX29* transcript accumulation. As the fruit matures, the transcript abundance of both these *SlBBX* genes decreases. Once ripening initiates, the conversion of chloroplast to chromoplast begins and SlRIN accumulates, activating the expression of several ripening associated genes, including *SlBBX19*, *SlBBX20* and *SlBBX26*. During ripening, these three *SlBBX* genes are also positively regulated by SlPHYs, probably, through the repression of several light signalling negative regulators, such as COP1 and PIFs. The absence of properly differentiated chloroplast due to SlGLK2 deficiency attenuates the upregulation of *SlBBX19*, *SlBBX20* and *SlBBX26* during ripening. Continuous lines indicate direct effect; dotted lines indicate that the effects may not be due to direct interaction. Arrow-ended lines indicate induction; bar-ended lines indicate repression.

Collectively, data obtained here provided a robust phylogenetic analysis of BBX proteins, giving a new perspective of the events that led to the diversification of these proteins in six structure groups. A comprehensive transcriptional profile of 15 *SlBBX*s revealed a correlation of mRNA amounts with the state of chloroplast development, as well as their regulation by the light signalling cascade. Additionally, a more detailed profiling in fruits led to the identification of three putative SlRIN-regulated ripening-associated *SlBBX* genes and other three loci associated with the early fruit development (Fig. 6). These results give insights on putative roles of SlBBX proteins in other light-regulated physiological process aside seedling photomorphogenesis and allow the identification of putative candidates for further characterization that may affect tomato fruit development and/or ripening.

## Supplementary information

Supplementary Information.
