# Supplementary material for: Light and ripening-regulated BBX protein-encoding genes in Solanum lycopersicum
Source: Sci Rep. 2020 Nov 6;10:19235. doi: 10.1038/s41598-020-76131-0 (PMC7648751; doi:10.1038/s41598-020-76131-0)
Supplement: Supplementary file 1 — Supplementary Information. [file 41598_2020_76131_MOESM1_ESM.pdf]

Supplementary material

Light and ripening-regulated B-BOX protein-encoding genes in *Solanum lycopersicum*

Bruno Silvestre Lira, Maria José Oliveira, Lumi Shiose, Raquel Tsu Ay Wu, Daniele Rosado, Alessandra Cavalcanti Duarte Lupi, Luciano Freschi, Magdalena Rossi

a

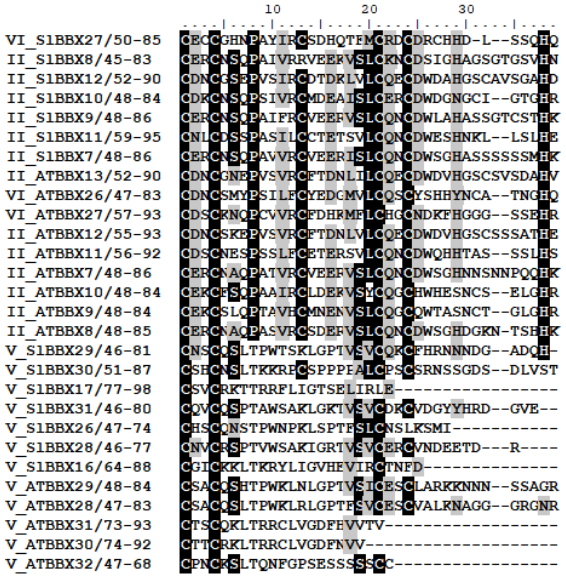

b

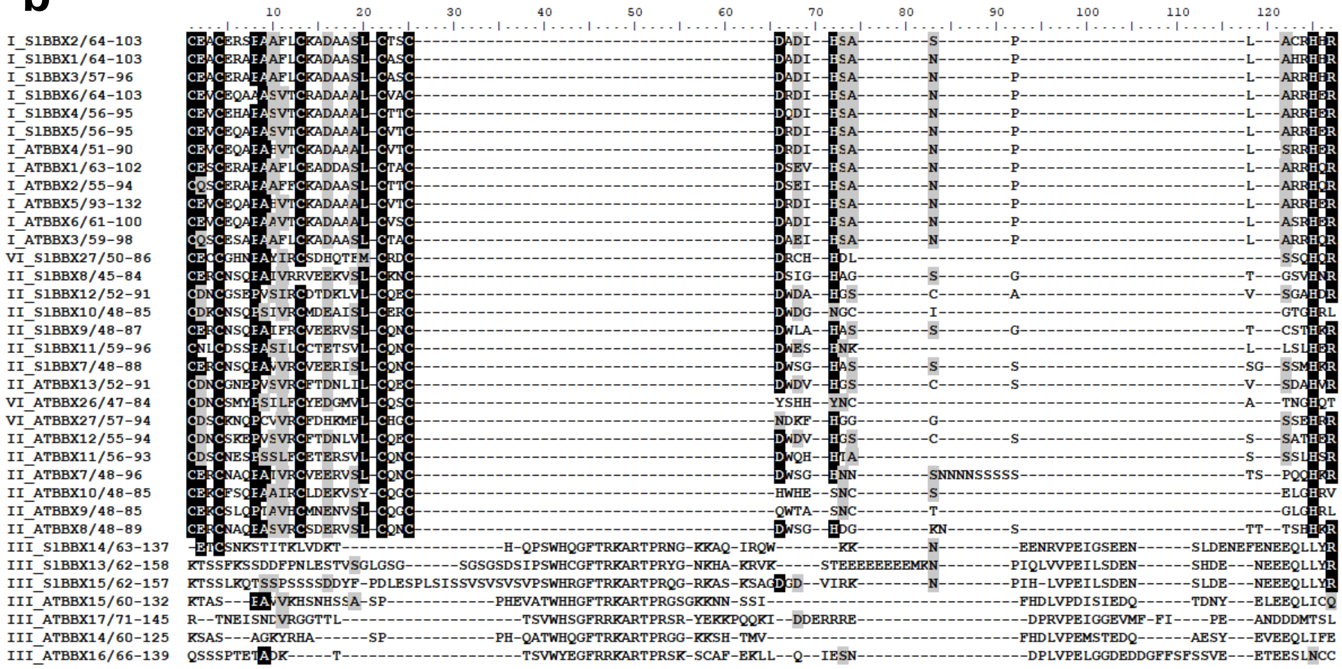

Supplementary Figure S1. Detail of BBX2 domain of structure. Proteins sequences of the members of structure group II (including structure group VI) and V (**a**) and of structure groups I, II and III (**b**) were aligned by Tcoffee Expresso package. The BBX2 domain is detailed, showing conserved residues that can be still found in structure group V proteins, however none can be identified in structure group III. Threshold for shading: 50%.

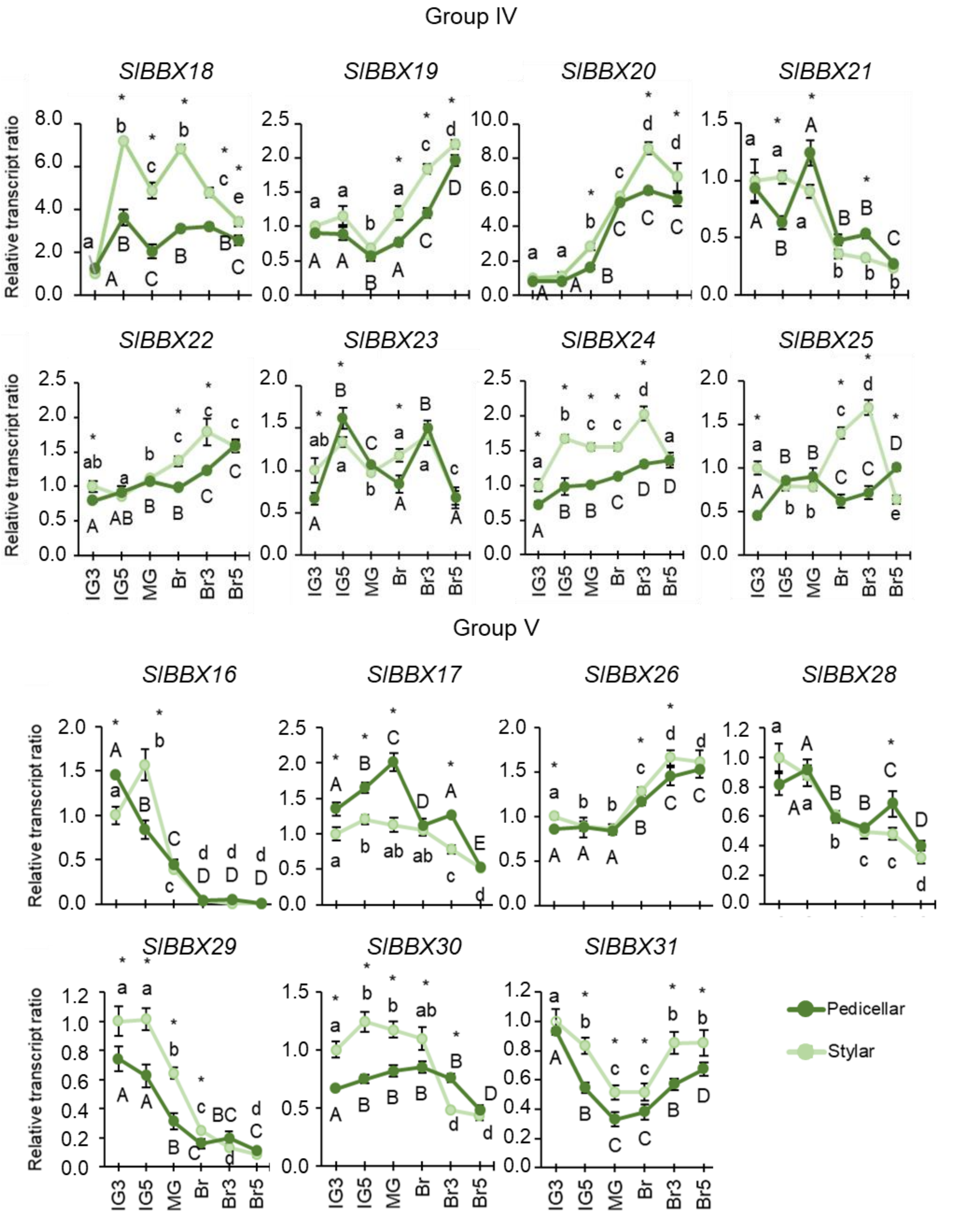

Supplementary Figure S2. Transcript profile of group IV and V SlBBX genes throughout fruit development and ripening. Fruits were sectioned in pedicellar and stylar portions. Two immature green (IG3 and IG5), mature green (MG) and three ripening stages (Br, breaker; Br3, 3 days after Br; Br5, 5 days after Br) were sampled for the analysis. Data were normalised against the stylar IG3 sample. Values are means ± SE of at least three biological replicates. Different letters indicate statistically significant differences between fruit stages within each portion, lower case (stylar portion) and upper case (pedicellar portion). Asterisks indicate statistically significant differences in a given stage between both portions.

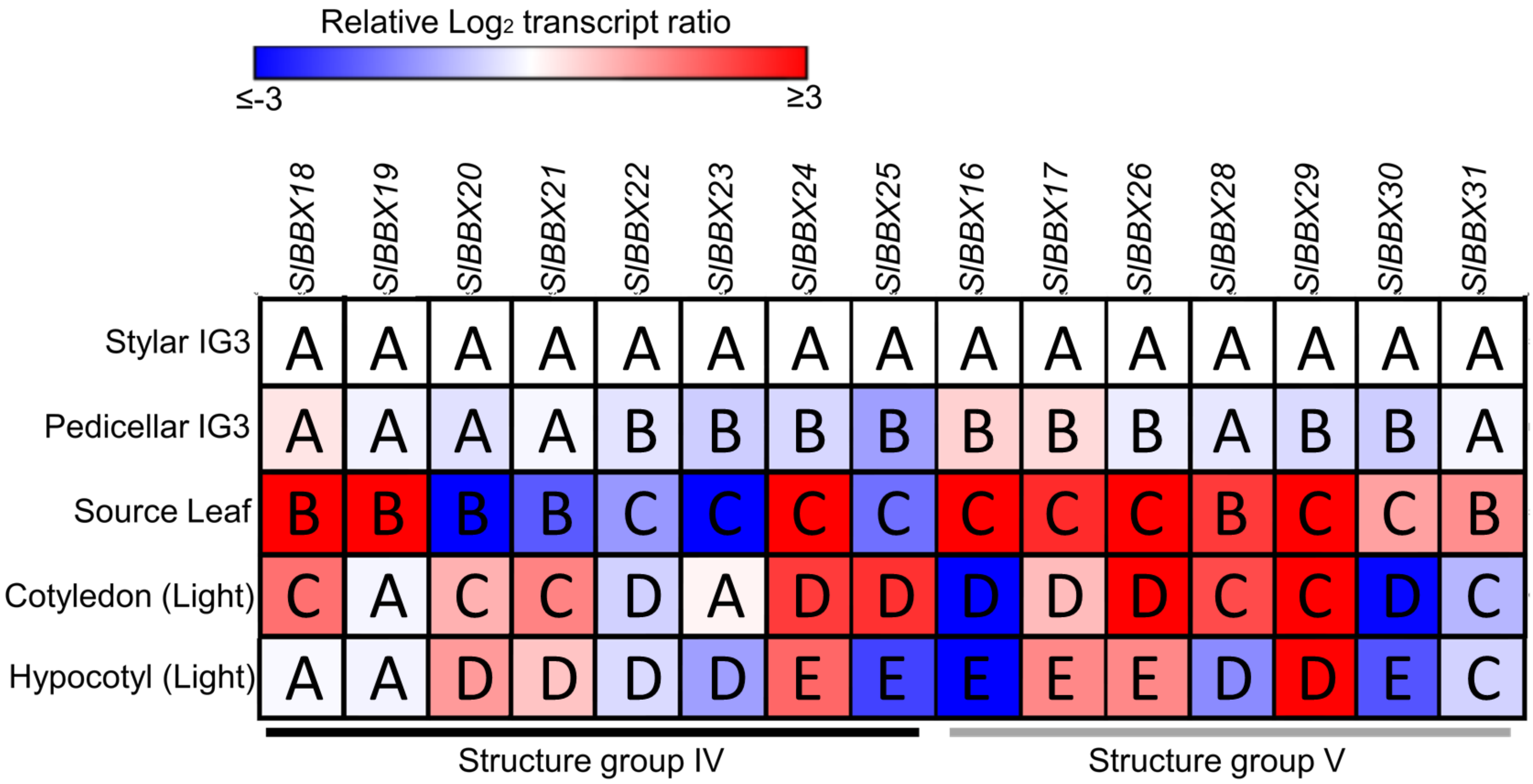

Supplemental Figure S3. Relative mRNA abundance of structure groups IV and V *SlBBXs* among tomato organs. Heatmap representation of the relative transcript ratio of each *SlBBX* among the four organs assayed. Data were normalysed against the stylar IG3 sample. Values are means of at least three biological replicates. Different letters indicate statistical significant differences between organs.

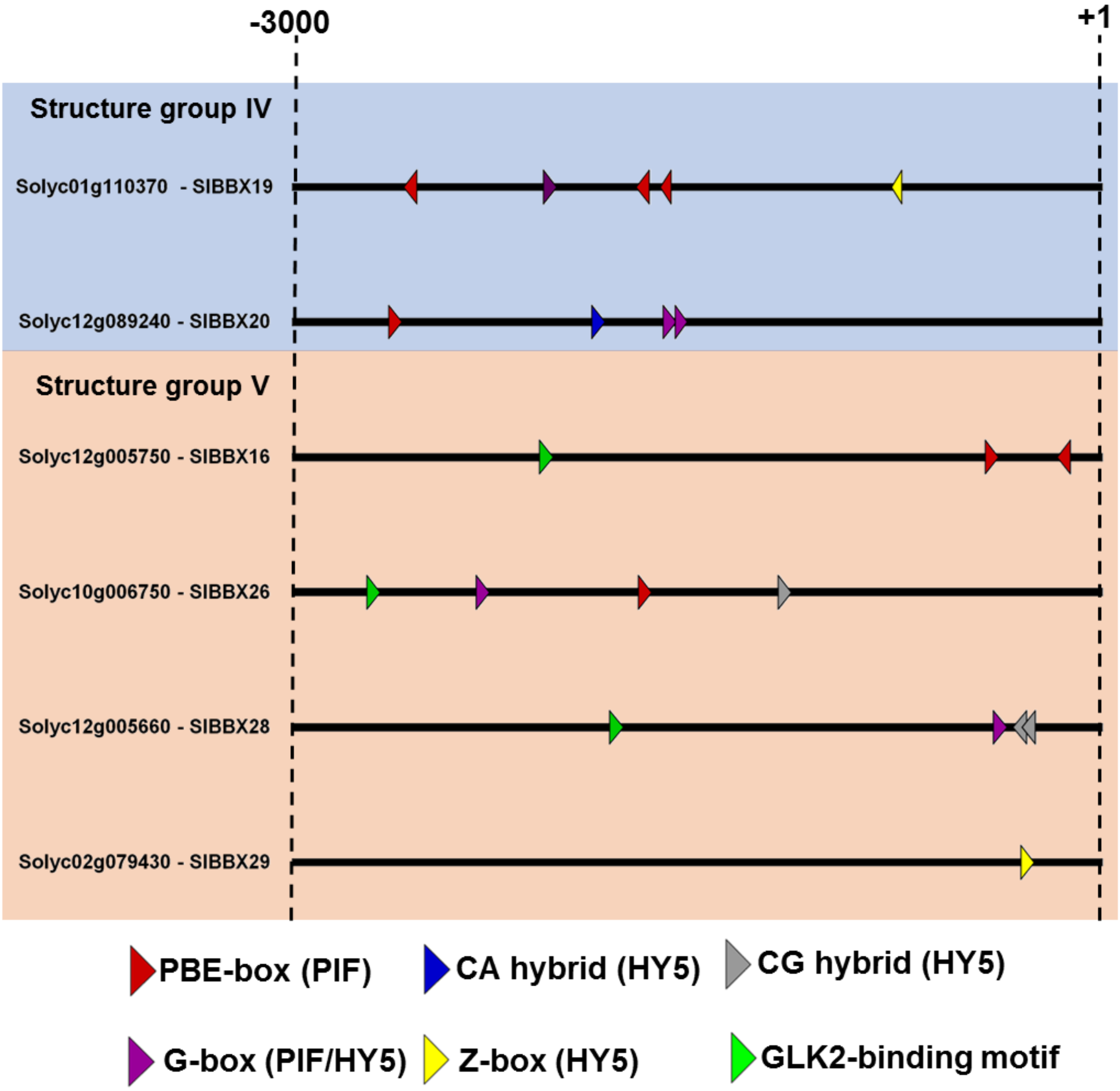

Supplementary Figure S4. Analysis of SlBBX encoding gene promoters. For each gene, 3000 bp upstrean of the transcription initiation site were retrieved and scanned for PBE-box (CACATG, Zhang et al. 2013), CA (GACGTA) and CG (GACGTG) hybrid motifs (Lee et al. 2007), G-box (CACGTG, Zhang et al. 2013), Z-box (ATACGTGT, Yadav et al. 2002) and GLK2-binding motif (CCAATC, Waters et al. 2013).

Suplementary Table S1. Sequences used for the phylogenetic reconstructions

Locus	*Species*	Sequence name	Structure Group
AT2G24790	*Arabidopsis thaliana*	AtBBX4	I
AT3G02380	*Arabidopsis thaliana*	AtBBX3	I
AT5G15840	*Arabidopsis thaliana*	AtBBX1	I
AT5G15850	*Arabidopsis thaliana*	AtBBX2	I
AT5G24930	*Arabidopsis thaliana*	AtBBX5	I
AT5G57660	*Arabidopsis thaliana*	AtBBX6	I
AT1G28050	*Arabidopsis thaliana*	AtBBX13	II
AT2G33500	*Arabidopsis thaliana*	AtBBX12	II
AT2G47890	*Arabidopsis thaliana*	AtBBX11	II
AT3G07650	*Arabidopsis thaliana*	AtBBX7	II
AT3G21880	*Arabidopsis thaliana*	AtBBX10	II
AT4G15250	*Arabidopsis thaliana*	AtBBX9	II
AT5G48250	*Arabidopsis thaliana*	AtBBX8	II
AT1G25440	*Arabidopsis thaliana*	AtBBX15	III
AT1G49130	*Arabidopsis thaliana*	AtBBX17	III
AT1G68520	*Arabidopsis thaliana*	AtBBX14	III
AT1G73870	*Arabidopsis thaliana*	AtBBX16	III
AT1G06040	*Arabidopsis thaliana*	AtBBX24	IV
AT1G75540	*Arabidopsis thaliana*	AtBBX21	IV
AT1G78600	*Arabidopsis thaliana*	AtBBX22	IV
AT2G21320	*Arabidopsis thaliana*	AtBBX18	IV
AT2G31380	*Arabidopsis thaliana*	AtBBX25	IV
AT4G10240	*Arabidopsis thaliana*	AtBBX23	IV
AT4G38960	*Arabidopsis thaliana*	AtBBX19	IV
AT4G39070	*Arabidopsis thaliana*	AtBBX20	IV
AT3G21150	*Arabidopsis thaliana*	AtBBX32	V
AT3G21890	*Arabidopsis thaliana*	AtBBX31	V
AT4G15248	*Arabidopsis thaliana*	AtBBX30	V
AT4G27310	*Arabidopsis thaliana*	AtBBX28	V
AT5G54470	*Arabidopsis thaliana*	AtBBX29	V
AT1G60250	*Arabidopsis thaliana*	AtBBX26	VI
AT1G68190	*Arabidopsis thaliana*	AtBBX27	VI
Cre06.g278159	*Chlamydomonas reinhardtii*		I/II/III/V-like
Cre12.g534100	*Chlamydomonas reinhardtii*		I/II/III/V-like
XP_005852267	*Chlorella variabilis*		I/II/III/V-like
XP_005652299	*Coccomyxa subellipsoidea C-169*		I/II/III/V-like
XP_005652347	*Coccomyxa subellipsoidea C-169*		I/II/III/V-like
NP_000372	*Homo sapiens*		Human
NP_001034200	*Homo sapiens*		Human
NP_005753	*Homo sapiens*		Human
NP_055978	*Homo sapiens*		Human
NP_056989	*Homo sapiens*		Human
NP_056990	*Homo sapiens*		Human
NP_112223	*Homo sapiens*		Human
NP_150241	*Homo sapiens*		Human
NP_258411	*Homo sapiens*		Human
NP_438112	*Homo sapiens*		Human
NP_689829	*Homo sapiens*		Human

NP_997279	*Homo sapiens*		Human
XP_011540501	*Homo sapiens*		Human
XP_016865110	*Homo sapiens*		Human
XP_002501202	*Micromonas commoda*		I/II/III/V-like
XP_002503322	*Micromonas commoda*		I/II/III/V-like
XP_002504098	*Micromonas commoda*		I/II/III/V-like
XP_002506129	*Micromonas commoda*		I/II/III/V-like
XP_002501766	*Micromonas commoda*		IV-like
XP_003056761	*Micromonas pusilla CCMP1545*		I/II/III/V-like
XP_003059624	*Micromonas pusilla CCMP1545*		I/II/III/V-like
XP_003058534	*Micromonas pusilla CCMP1545*		IV-like
XP_001417480	*Ostreococcus lucimarinus CCE9901*		I/II/III/V-like
XP_001417557	*Ostreococcus lucimarinus CCE9901*		I/II/III/V-like
XP_001419738	*Ostreococcus lucimarinus CCE9901*		I/II/III/V-like
XP_001419373	*Ostreococcus lucimarinus CCE9901*		IV-like
XP_003078900	*Ostreococcus tauri*		I/II/III/V-like
XP_003081141	*Ostreococcus tauri*		I/II/III/V-like
XP_022839368	*Ostreococcus tauri*		I/II/III/V-like
XP_022840999	*Ostreococcus tauri*		IV-like
Solyc02g089500	*Solanum lycopersicum*	SlBBX2	I
Solyc02g089520	*Solanum lycopersicum*	SlBBX1	I
Solyc02g089540	*Solanum lycopersicum*	SlBBX3	I
Solyc07g006630	*Solanum lycopersicum*	SlBBX6	I
Solyc08g006530	*Solanum lycopersicum*	SlBBX4	I
Solyc12g096500	*Solanum lycopersicum*	SlBBX5	I
Solyc05g020020	*Solanum lycopersicum*	SlBBX8	II
Solyc05g024010	*Solanum lycopersicum*	SlBBX12	II
Solyc05g046040	*Solanum lycopersicum*	SlBBX10	II
Solyc07g045180	*Solanum lycopersicum*	SlBBX9	II
Solyc09g074560	*Solanum lycopersicum*	SlBBX11	II
Solyc12g006240	*Solanum lycopersicum*	SlBBX7	II
Solyc03g119540	*Solanum lycopersicum*	SlBBX14	III
Solyc04g007210	*Solanum lycopersicum*	SlBBX13	III
Solyc05g009310	*Solanum lycopersicum*	SlBBX15	III
Solyc01g110180	*Solanum lycopersicum*	SlBBX25	IV
Solyc01g110370	*Solanum lycopersicum*	SlBBX19	IV
Solyc02g084420	*Solanum lycopersicum*	SlBBX18	IV
Solyc04g081020	*Solanum lycopersicum*	SlBBX21	IV
Solyc06g073180	*Solanum lycopersicum*	SlBBX24	IV
Solyc07g062160	*Solanum lycopersicum*	SlBBX22	IV
Solyc12g005420	*Solanum lycopersicum*	SlBBX23	IV
Solyc12g089240	*Solanum lycopersicum*	SlBBX20	IV
Solyc02g079430	*Solanum lycopersicum*	SlBBX29	V
Solyc06g063280	*Solanum lycopersicum*	SlBBX30	V
Solyc07g052620	*Solanum lycopersicum*	SlBBX17	V
Solyc07g053140	*Solanum lycopersicum*	SlBBX31	V
Solyc10g006750	*Solanum lycopersicum*	SlBBX26	V
Solyc12g005660	*Solanum lycopersicum*	SlBBX28	V
Solyc12g005750	*Solanum lycopersicum*	SlBBX16	V
Solyc04g007470	*Solanum lycopersicum*	SlBBX27	VI

Vocar.0002s0569	*Volvox carteri*	I/II/III/V-like
XP_002946907	*Volvox carteri f. nagariensis*	I/II/III/V-like

Suplementary Table S2. Primers used in the experiments

Gene name	Locus	Experiment	Primer sequence (5' - 3')
SlBBX16	Solyc12g005750	RT-qPCR	F: GCCTATGTATATTGTGAAGCAGATAATG<br>R: GTGAAACTCCAATCAAATACCTC
SlBBX17	Solyc07g052620	RT-qPCR	F: GTGGAAGAAGAGAGGGTGAC<br>R: AATCGTCGGCAAAATCAGC
SlBBX18	Solyc02g084420	RT-qPCR	F: GGGTGAGTTGTGGTAGAAATG<br>R: CGGTATCTAATGTCTTCCTGGC
SlBBX19	Solyc01g110370	RT-qPCR	F: CCAACCAAACAGGGAGACTGC<br>R: CATCAGGAACCACGCCGACAG
		ChIP RT-qPCR	F: GGACCCGAAAGAGAAAGG<br>R: GCTGTTGAAGATTTTATTGATACACAAGATTG
SlBBX20	Solyc12g089240	RT-qPCR	F: CTCTGCTCTCTACTCTGCTCC<br>R: GATGATACTGCTCCTCCCTTAG
		ChIP RT-qPCR	F: GAGAATATCTAATTGTGAAGTCAATAGC<br>R: CATATTGGAATGTGTATATCGTTTGAC
SlBBX21	Solyc04g081020	RT-qPCR	F: AGAGATGCTACCTGGATGGC<br>R: AGAAGATGATGATGGGCTGATTTG
SlBBX22	Solyc07g062160	RT-qPCR	F: GACATCACAGTCCATTCAGAAGG<br>R: CTCTCCACCAAGTTTGCCAT
SlBBX23	Solyc12g005420	RT-qPCR	F: GGATAGGGCTTTGCTGTGC<br>R: GACTTTCGTGAGGATGCTGATGC
SlBBX24	Solyc06g073180	RT-qPCR	F: ACTGTGATTTGCTGTGCTGATG<br>R: GCTCAAGGCTACACGGATTC
SlBBX25	Solyc01g110180	RT-qPCR	F: GATGTTTGTGATAAAGAAGAGG<br>R: CTCCACTTAGAAGAAATCTGTTGTG
SlBBX26	Solyc10g006750	RT-qPCR	F: ATTGTGAGTTATGTGGTGAGAAC<br>R: CGGCGACGGCGAGGGAGAGAAATC
		ChIP RT-qPCR	F: GCCCATACTCCAAAGATAGATATG<br>R: AGTTCTAGTCACATTTGCTTAC
SlBBX28	Solyc12g005660	RT-qPCR	F: GACAGAGAAGAGGAAGAGAAGG<br>R: CCACCGTCGCTGAACATC
SlBBX29	Solyc02g079430	RT-qPCR	F: GGTCCCACTGTTTCTGTTTG<br>R: CATCATCTTCTTCTTCTTCCG
SlBBX30	Solyc06g063280	RT-qPCR	F: GGACAGCGACTCTAACGG<br>R: GAGGCAGACCCCTAAACC
SlBBX31	Solyc07g053140	RT-qPCR	F: GTTTGTGTTGGGACTGTGATG<br>R: GGTGGAGGCGTCGTATTTGAC
SlRIN	Solyc05g012020	ChIP Cloning	F: CACCATGGGTAGAGGGAAAGTAGAATTG<br>R: AAGCATCCATCCAGGTAC
ACTIN4	Solyc04g011500	ChIP RT-qPCR	F: CCTTCCACATGCCATTCTCC<br>R: CCACGCTCGGTCAGGATCT
EXPRESSED	Solyc07g025390	RT-qPCR	F: GCTAAGAACGCTGGACCTAATG<br>R: TGGGTGTGCCTTTCTGAATG
TIP41	Solyc10g049850	RT-qPCR	F: ATGGAGTTTTTGAGTCTTCTGC<br>R: GCTGCGTTTCTGGCTTAGG

Supplementary Table S3. Transcript profile of group IV and V *SlBBX* genes in leaves and seedlings

		Group IV							
Leaves		*SlBBX18*	*SlBBX19*	*SlBBX20*	*SlBBX21*	*SlBBX22*	*SlBBX23*	*SlBBX24*	*SlBBX25*
Sink		1.00 ± 0.06	1.00 ± 0.08	1.00 ± 0.07	1.00 ± 0.12	1.00 ± 0.06	1.00 ± 0.18	1.00 ± 0.07	1.00 ± 0.26
Source		**1.53 ± 0.09**	**2.86 ± 0.21**	**5.57 ± 0.19**	**1.43 ± 0.1**	**1.75 ± 0.13**	**1.77 ± 0.2**	**2.38 ± 0.15**	0.91 ± 0.09
Seedlings		*SlBBX18*	*SlBBX19*	*SlBBX20*	*SlBBX21*	*SlBBX22*	*SlBBX23*	*SlBBX24*	*SlBBX25*
Hypocotyl	Dark	1.00 ± 0.05 A	1.00 ± 0.06 A	1.00 ± 0.03 A	1.00 ± 0.04 A	1.00 ± 0.05 A	1.00 ± 0.06 A	1.00 ± 0.05 A	1.00 ± 0.08 A
	Light	2.89 ± 0.09 B	1.01 ± 0.03 A	1.01 ± 0.05 A	0.43 ± 0.03 B	0.89 ± 0.04 A	0.93 ± 0.07 A	1.70 ± 0.11 B	0.46 ± 0.09 B
Cotyledon	Dark	5.36 ± 0.08 C	1.42 ± 0.05 B	2.28 ± 0.07 B	0.48 ± 0.07 B	1.26 ± 0.08 B	2.43 ± 1.09 B	2.34 ± 0.02 C	42.96 ± 0.07 C
	Light	9.78 ± 0.05 D	1.03 ± 0.09 A	0.83 ± 0.09 C	1.01 ± 0.07 A	0.83 ± 0.29 A	2.30 ± 0.93 B	2.51 ± 0.03 C	12.76 ± 0.08 D

		Group V						
Leaves		*SlBBX16*	*SlBBX17*	*SlBBX26*	*SlBBX28*	*SlBBX29*	*SlBBX30*	*SlBBX31*
Sink		1.00 ± 0.12	1.00 ± 0.07	1.00 ± 0.05	1.00 ± 0.03	1.00 ± 0.16	1.00 ± 0.05	1.00 ± 0.04
Source		**4.44 ± 0.16**	**3.05 ± 0.25**	**3.21 ± 0.33**	**2.02 ± 0.21**	**2.86 ± 0.11**	1.04 ± 0.16	**2.44 ± 0.25**
Seedlings		*SlBBX16*	*SlBBX17*	*SlBBX26*	*SlBBX28*	*SlBBX29*	*SlBBX30*	*SlBBX31*
Hypocotyl	Dark	1.00 ± 0.1 A	1.00 ± 0.01 A	1.00 ± 0.06 A	1.00 ± 0.09 A	1.00 ± 0.04 A	1.00 ± 0.09 A	1.00 ± 0.03 A
	Light	1.24 ± 0.12 A	3.63 ± 0.06 B	0.64 ± 0.12 B	1.94 ± 0.23 B	0.92 ± 0.09 A	4.29 ± 0.12 B	1.18 ± 0.07 A
Cotyledon	Dark	0.12 ± 0.47 B	0.99 ± 0.09 A	4.17 ± 0.51 C	4.32 ± 0.17 C	0.81 ± 0.08 B	2.92 ± 0.14 C	0.76 ± 0.06 B
	Light	0.44 ± 0.34 C	2.05 ± 0.05 C	7.31 ± 0.19 D	7.15 ± 0.49 D	1.33 ± 0.09 C	2.30 ± 0.26 C	0.98 ± 0.08 A

Values are normalised against the respective sink leaf sample and dark-hypocotyl and represent mean ± SE of at least three biological replicates. For leaves, statistically significant differences to the corresponding normaliser sample are in bold.For seedling, statistically significant differences among the samples are represented with different letters.

Supplementary Table S4. Transcript profile of group IV and V *SlBBX* genes in the pedicellar (top) portion throughout fruit development and ripening

	Group IV															
	SlBBX18		*SlBBX19*		*SlBBX20*		*SlBBX21*		*SlBBX22*		*SlBBX23*		*SlBBX24*		*SlBBX25*	
Immature green 3	1.00 ± 0.14	a	1.00 ± 0.04	a	1.00 ± 0.10	a	1.00 ± 0.14	a	1.00 ± 0.03	a	1.00 ± 0.41	a	1.00 ± 0.06	a	1.00 ± 0.32	a
Immature green 5	2.95 ± 0.30	b	0.99 ± 0.11	a	1.03 ± 0.03	a	0.68 ± 0.06	b	1.16 ± 0.11	ab	2.43 ± 0.64	b	1.36 ± 0.25	b	1.88 ± 0.43	b
Mature green	1.65 ± 0.25	c	0.63 ± 0.02	b	2.07 ± 0.14	b	1.33 ± 0.12	a	1.36 ± 0.06	b	1.61 ± 0.04	c	1.39 ± 0.04	b	1.98 ± 0.26	b
Breaker	2.52 ± 0.11	b	0.85 ± 0.06	a	6.96 ± 0.17	c	0.51 ± 0.05	b	1.23 ± 0.02	b	1.27 ± 0.31	a	1.56 ± 0.04	c	1.36 ± 0.34	c
Breaker +3	2.59 ± 0.04	b	1.33 ± 0.08	c	7.79 ± 0.28	c	0.57 ± 0.04	b	1.55 ± 0.05	c	2.27 ± 0.28	b	1.81 ± 0.06	d	1.58 ± 0.17	c
Breaker +5	2.08 ± 0.10	c	2.19 ± 0.09	d	7.13 ± 0.47	c	0.29 ± 0.01	c	2.00 ± 0.12	c	1.02 ± 0.12	a	1.88 ± 0.10	d	2.21 ± 0.08	d

	Group V													
	SlBBX16		*SlBBX17*		*SlBBX26*		*SlBBX28*		*SlBBX29*		*SlBBX30*		*SlBBX31*	
Immature green 3	1.00 ± 0.01	a	1.00 ± 0.07	a	1.00 ± 0.03	a	1.00 ± 0.21	a	1.00 ± 0.16	a	1.00 ± 0.04	a	1.00 ± 0.02	a
Immature green 5	0.58 ± 0.07	b	1.22 ± 0.13	b	1.02 ± 0.13	a	1.12 ± 0.08	a	0.84 ± 0.19	a	1.12 ± 0.06	b	0.59 ± 0.06	b
Mature green	0.31 ± 0.04	c	1.49 ± 0.22	c	0.97 ± 0.04	a	0.72 ± 0.04	b	0.42 ± 0.08	b	1.23 ± 0.04	b	0.36 ± 0.02	c
Breaker	0.03 ± 0.00	d	0.83 ± 0.17	d	1.35 ± 0.04	b	0.63 ± 0.02	b	0.22 ± 0.05	c	1.28 ± 0.08	b	0.41 ± 0.03	c
Breaker +3	0.03 ± 0.00	d	0.94 ± 0.03	a	1.69 ± 0.12	c	0.79 ± 0.13	c	0.27 ± 0.01	c	1.14 ± 0.06	b	0.61 ± 0.02	b
Breaker +5	0.01 ± 0.00	d	0.39 ± 0.02	e	1.78 ± 0.19	c	0.51 ± 0.04	d	0.15 ± 0.02	c	0.73 ± 0.06	c	0.72 ± 0.04	d

Values are normalised against the respective immature green 3 sample and represent mean ± SE of at least three biological replicates. Statistically significant differences among the samples are represented with different letters.

Supplementary Table S5. Relative transcript ratio of *SlBBXs* in fruit specific light perception or chloroplast deficient tomato lines

	SlBBX16	*SlBBX19*	*SlBBX20*	*SlBBX26*	*SlBBX28*	*SlBBX29*
Immature green 3						
WT	1.00 ± 0.31	1.00 ± 0.50	1.00 ± 0.25	1.00 ± 0.17	1.00 ± 0.05	1.00 ± 0.42
Slglk2	1.48 ± 0.26	0.54 ± 0.09	**1.90 ± 0.10**	**1.74 ± 0.07**	0.91 ± 0.08	**0.23 ± 0.04**
PPC2::SlPHYA- RNAi	**2.22 ± 0.44**	0.66 ± 0.23	1.02 ± 0.04	**2.98 ± 0.35**	0.93 ± 0.14	0.52 ± 0.31
PPC2::SlPHYB2- RNAi	**2.97 ± 0.51**	0.75 ± 0.02	1.15 ± 0.14	**1.50 ± 0.11**	1.09 ± 0.08	0.46 ± 0.12
Mature green						
WT	1.00 ± 0.14	1.00 ± 0.09	1.00 ± 0.54	1.00 ± 0.10	1.00 ± 0.10	1.00 ± 0.49
Slglk2	**0.45 ± 0.03**	**0.68 ± 0.04**	0.89 ± 0.09	0.72 ± 0.07	0.50 ± 0.21	1.85 ± 0.40
PPC2::SlPHYA- RNAi	**0.60 ± 0.09**	**1.59 ± 0.20**	1.15 ± 0.13	0.87 ± 0.23	0.96 ± 0.09	**0.22 ± 0.08**
PPC2::SlPHYB2- RNAi	1.33 ± 0.12	0.87 ± 0.47	2.13 ± 0.20	0.99 ± 0.04	0.87 ± 0.18	**5.82 ± 0.91**
Breaker						
WT	1.00 ± 0.09	1.00 ± 0.37	1.00 ± 0.15	1.00 ± 0.05	1.00 ± 0.09	1.00 ± 0.07
Slglk2	0.44 ± 0.29	0.97 ± 0.16	0.72 ± 0.38	**0.56 ± 0.10**	0.70 ± 0.19	0.57 ± 0.34
PPC2::SlPHYA- RNAi	**3.76 ± 0.54**	1.20 ± 0.12	1.03 ± 0.08	0.78 ± 0.17	**0.57 ± 0.01**	1.13 ± 0.03
PPC2::SlPHYB2- RNAi	**6.55 ± 2.26**	1.09 ± 0.22	0.99 ± 0.13	**0.44 ± 0.03**	**1.95 ± 0.74**	**6.28 ± 2.02**
Breaker +3						
WT	1.00 ± 0.34	1.00 ± 0.06	1.00 ± 0.20	1.00 ± 0.13	1.00 ± 0.13	1.00 ± 0.42
Slglk2	**0.15 ± 0.04**	**0.29 ± 0.04**	**0.50 ± 0.45**	0.43 ± 0.13	1.02 ± 0.16	1.27 ± 0.12
PPC2::SlPHYA- RNAi	**0.38 ± 0.06**	0.50 ± 0.01	**0.53 ± 0.35**	**0.31 ± 0.06**	0.70 ± 0.09	1.07 ± 0.32
PPC2::SlPHYB2- RNAi	**0.09 ± 0.03**	**0.23 ± 0.01**	**0.36 ± 0.19**	**0.26 ± 0.03**	0.53 ± 0.13	0.78 ± 0.12
Breaker +5						
WT	1.00 ± 0.27	1.00 ± 0.06	1.00 ± 0.32	1.00 ± 0.20	1.00 ± 0.31	1.00 ± 0.42
Slglk2	**3.43 ± 1.04**	0.70 ± 0.32	0.69 ± 1.51	0.81 ± 0.24	**3.14 ± 0.24**	0.78 ± 0.25
PPC2::SlPHYA- RNAi	**0.48 ± 0.04**	**0.24 ± 0.01**	**0.28 ± 0.35**	1.25 ± 0.16	0.64 ± 0.19	**0.15 ± 0.01**
PPC2::SlPHYB2- RNAi	**0.23 ± 0.06**	**0.28 ± 0.11**	**0.22 ± 0.91**	**0.55 ± 0.11**	1.74 ± 0.22	0.52 ± 0.06

Values are normalised against the respective WT sample and represent mean ± SE of at least three biological replicates. Statistically significant values to the respective WT sample are in bold.
